# Changes of immune cells in patients with hepatocellular carcinoma treated by radiofrequency ablation and hepatectomy, a pilot study

**DOI:** 10.1515/biol-2021-0105

**Published:** 2021-09-18

**Authors:** Zusheng Yu, Guowei Li, Hang Yu, Tetsuya Asakawa

**Affiliations:** Department of Hepatobiliary Surgery, The First People’s Hospital of Fuyang Hangzhou, Hangzhou, 311400, China; Department of Gastrointestinal Surgery, The First Affiliated Hospital, School of Medicine, Zhejiang University, Hangzhou, 310003, China; Department of Neurology, The Eighth Affiliated Hospital, Sun Yat-Sen University, Shennanzhong Road 3025, Shenzhen, Guangdong·Province, 518033, China; Department of Neurosurgery, Hamamatsu University School of Medicine, Handayama, Hamamatsu-city, Shizuoka, 4313192, Japan; Research Base of Traditional Chinese Medicine Syndrome, Fujian University of Traditional Chinese Medicine, Fuzhou 350122, China

**Keywords:** small HCC, RFA, hepatectomy, immune cells, PBMCs

## Abstract

In this pilot study, we compared the dynamic changes of circulating immune cells between patients with hepatocellular carcinoma (HCC) who underwent radiofrequency ablation (RFA) and hepatectomy. Seventy-three patients were enrolled in this study. Flow cytometry assay was performed to determine the immune cells in the peripheral blood mononuclear cells (PBMCs) before treatment and on days 7, 14, and 28 after treatment. We found that in the RFA group, the circulating cluster of differentiation (CD)4+ cells, the CD4+/CD8+ ratio, and natural killer (NK) cells continued to increase, and the circulating CD8+ cells continued to decrease after the treatment. In contrast, in the surgery group, the circulating CD4+ cells and CD4+/CD8+ ratio decreased over the first seven postoperative days and then began to increase, and CD8+ cells decreased on the first 7 postoperative days and began to increase thereafter. The changes of immune cells in tumor tissues consisted of an increase in the number of CD4+ cells, CD8+ cells, CD3+ cells, and NK cells immediately after RFA. Our results show that postoperative immune function continued to improve after RFA, but after surgery, it decreased in the first week and started to improve thereafter. These findings are important for clinicians when selecting the appropriate therapy for HCC.

## 1 Introduction

Hepatocellular carcinoma (HCC) is the most common malignant tumor of the liver. According to data in 2018, it is in the top six most frequent cancers and the fourth cause of tumor-related deaths [1]. HCC is not rare in China because of the high prevalence of hepatitis B virus (HBV) infection. It has been estimated that the HBV infection prevalence is approximately 6–10% in the north of China and over 10% in the south of China [2]. In China, HCC is ranked fourth on the list of diagnosed cancers and the fourth cause of tumor-related deaths [3]. It is considered a global public health concern. Unfortunately, although hepatectomy with liver transplantation (HLC) is the potentially effective treatment for HCC, less than 20% of patients with HCC have the possibility of undergoing HLC [4]. Many locoregional therapies for HCC, particularly small HCC (SHCC) therapies such as radiofrequency ablation (RFA), transcatheter arterial chemoembolization, and microwave coagulation ablation, have been developed. It is well documented that RFA is a safe and effective therapy for SHCC [5, 6]. Chen et al. recently reported that, in patients treated by RFA, progression-free survival time and 5-year survival rate are 25.0  ±  22.7 months (median: 17.0 months) and 28.5%, respectively [6]. However, the underlying mechanisms associated with the treatment by RFA are not fully understood. Some authors consider necrosis and apoptosis to be the underlying mechanisms [7]. The biological behavior of HCC is remarkably affected by the tumor immune micro-environment [8]. T lymphocytes play a crucial role in the development and progression of HCC, and cluster of differentiation (CD)4+ T helper (Th) cells play a central role in orchestrating host immune responses. Th1 cells produce cytokines such as interferon-γ to trigger antimicrobial and antitumor cytotoxic responses, whereas other Th cells suppress T cell immunity in physiological and pathological conditions [9]. It is known that CD8+ T cells play a cytotoxic role in antitumor immune reactions by releasing granules such as perforin and granzymes [10]. CD8+ T cells are associated with tumor progression. Natural killer (NK) cells are potent effectors of the innate immune system, form the first line of defense against malignancies, and are associated with high clinical survival and low recurrence rate [11]. Understanding the immunological mechanisms of RFA is necessary for the better application of RFA. However, there are limited studies on this subject matter. Sugimoto et al. compared postoperative immune responses between patients who underwent RFA and patients who underwent irreversible electroporation. They found no significant changes in peripheral blood mononuclear cells (PBMCs), namely, T cells, CD4+ cells, CD8+ T cells, and NK cells at preoperation, postoperation, postoperative day 1, and postoperative day 4 [12]. We hypothesized that, over a period longer than postoperative day 4, the observed changes in immune cells might increase.

In this study, we compared the changes of circulating immune cells between patients who underwent RFA and patients who underwent conventional hepatectomy. We observed changes of PBMCs over a longer postoperative period of 4 weeks. We also observed changes of immune cells in tumor tissues before and immediately after RFA. We aimed to determine the changes of immune cells that are due to RFA to enable a better understanding of the underlying immunological mechanisms associated with RFA.

## 2 Methods

### 2.1 Patients

A total of 73 patients with SHCC treated in our hospital from January 2012 to January 2016 were enrolled in this study. All the patients were pathologically diagnosed with SHCC based on prior liver biopsy. Patients with autoimmune diseases, patients undergoing immunosuppressant treatment or chemotherapy, patients with other systemic diseases including hematological and inflammatory diseases, and patients with poor compliance were excluded from this study. This study was rigorously designed, and the study protocol was explained to the patients and their relatives in detail.

**Informed consent:** Informed consent has been obtained from all individuals included in this study.**Ethical approval:** The research related to human use has been complied with all the relevant national regulations, institutional policies and in accordance with the tenets of the Helsinki Declaration, and has been approved by the ethics committee of the First People’s Hospital of Fuyang Hangzhou (approval number: 2020-003).

### 2.2 Experimental design

Fasting peripheral venous blood samples (10 mL) were collected from all enrolled patients at 7 am on the following time points: 1 day before treatment and at 7, 14, and 28 days after treatment. The blood samples were placed in heparin anticoagulant tubes and immediately sent for the subsequent experiments. A variant of percutaneous needle biopsy described by Hasegawa et al. [13] was used to obtain tumor tissues from patients who underwent RFA before and immediately after RFA.

Clinical evaluation was performed 1 month after treatment. Computed tomography and Ultrasonic B (EUB7500, Hitachi, Japan) scans were used to assess the operative state of each patient, and the Child–Pugh scale was used to evaluate liver function.

### 2.3 Treatments

A total of 38 patients underwent surgical resection (the surgery group). The surgical procedures were selected according to the criteria described in the previous study [14]. In brief, the state of ascites, serum total bilirubin level, and the indocyanine green retention rate at 15 min was assessed. During nonanatomic resection, liver parenchymal transection was performed 1–2 cm from the tumor surface. During anatomic resection, the following points were comprehensively considered: resection area, approach to the portal triad, and approaches to expose landmark vessels.

Thirty-five patients underwent RFA (the RFA group) according to the method described by Livraghi et al. [15]. RFA was performed under the guidance of color Doppler ultrasonography (EUB7500, Hitachi, Japan) at a probe frequency of 3.5–4.0 MHz. Retractable radiofrequency (RF) electrodes were connected to a 500 kHz RF generator (100 W, MSIS-1500, Medsphere, USA). The patient position was based on tumor location as detected on imaging examination. The electrodes were inserted according to the number, location, and size of the tumor under the guidance of Doppler ultrasonography. RFA was performed until the tumor was completely hyperechoic. RFA parameters were determined according to the state of each patient, and the duration of RFA was 8–12 min per course.

### 2.4 Flow cytometry assay for immune cells in PBMCs

The morphological experiments were performed as per the methods described in earlier studies [16, 17]. In brief, PBMCs were isolated by a Ficoll density gradient (TBD sciences, China). After suspension in phosphate-buffered saline (PBS), the PBMCs were counted using an automated cell viability analyzer (Vi-cellTMXR, Beckman Coulter). We then incubated the PBMCs with anti-CD3-FITC (11-0039), anti-CD4-PE-Cy7(25-0049), anti-CD8-APC (17-0088) monoclonal antibodies (mAbs), and anti-CD56-PE-Cy7 (25-0567, eBioscience, USA) for 30 min at 4°C. The appropriate isotype mAbs were used as controls. The percentage of positively stained cells was determined using a flow cytometer (LSR-II, BD Biosciences) equipped with BD FACSDiva software. For the analysis, at least 1 × 10^4^ lymphocytes were acquired.

### 2.5 Immunohistochemical staining of tumor tissues

Paraffin-embedded tumor tissue sections of 4 μm in thickness obtained from each patient were submitted for immunohistochemical staining. The samples were dewaxed and subjected to heat-induced epitope retrieval with a preheated epitope retrieval solution (10 mM citrate buffer, pH 6.0). Endogenous peroxidase activity was then blocked. The sections were incubated overnight at 4°C with one of the following primary mAbs: mouse anti-CD3, rabbit anti-CD8, mouse anti-CD56 (working solution; Zhongshan Golden Bridge Biotechnology, Beijing, China), or rabbit anti-CD4 (1:100; Leica, Wetzlar, Germany). After incubation with horse-radish peroxidase-conjugated secondary antibody (Invitrogen, Carlsbad, CA, USA) for 1 h at 37°C and development with diaminobenzidine, the sections were counterstained with hematoxylin. Negative control staining was performed with PBS instead of primary antibody. Images of 10 random areas of intratumoral regions were captured under 400× high power field (HPF; Olympus, Japan) ocular lens.

### 2.6 Statistical analysis

Data are presented as mean ± standard error of the mean. All statistical analyses were performed using SPSS software (V 19.00, IBM, IL, USA). The two-way analysis of variance was used for the comparison of quantitative data between the two groups. Mann–Whitney test was used for comparison of independent samples. Comparison of ratios was performed using the chi-square test. *p* values <0.05 were considered statistically significant.

## 3 Results

### 3.1 Clinical characteristics of the enrolled patients

The clinical characteristics are presented in Table 1. There were no significant differences in baseline clinical characteristics between the RFA and surgery groups.

**Table 1:** Clinical characteristics of enrolled patients

	RFA (*n* = 35)	Surgery (*n* = 38)
Age (years), median (IQR)	54 (51–64)	53 (49–63)
Gender (M/F)	23/12	24/14
Diagnosis	HCC21/SHC14	HCC29/SHC9
History of cirrhosis	18	26
Child–Pugh score	A13/B5	A24/B2
Tumor diameter (cm), median (IQR)	2.3 (2.1–3.8)	2.4 (2.2–4.1)

RFA: radiofrequency ablation; IQR: interquartile range.

RFA and surgery were satisfactorily performed. The types of liver resection are presented in Table 2. There was no incidence of surgical complications or postoperative death in any enrolled patient. Data of 1 month follow-up after treatment showed that there was no recurrence in any of the two groups. No significant artery lesion area enhancement was found in the RFA group. Tumor regional blood flows disappeared in 30 patients, and vascular resistance index decreased in five patients.

**Table 2:** Types of liver resection

	*n* (%)
Right hepatectomy	5 (13.2)
Left hepatectomy (segments II, III, IV)	7 (18.4)
Left lateral lobectomy (segments II, III)	5 (13.2)
Segment IV	1 (2.6)
Segment V	4 (10.5)
Atypical resection	16 (42.1)

### 3.2 Changes of circulating immune cells after RFA and surgery

**Figure 1:**
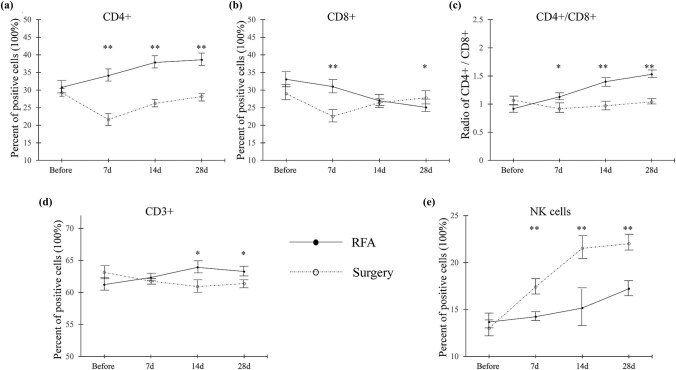
Circulating immune cells in the RFA and surgery groups. (a) CD4+ cells, (b) CD8+ cells, (c) CD4+/CD8+ ratio, (d) CD3+ cells, (e) NK cells. Percentage of cells that stained positively for the antibodies of interest were calculated. Data are presented as mean ± SEM; * means *p* < 0.05; ** means *p* < 0.01 (RFA versus surgery). RFA: radiofrequency ablation, CD: cluster of differentiation, NK: natural killer, SEM: standard error of the mean.

The dynamic changes of circulating immune cells after RFA and surgery are shown in Figure 1. The number of CD4+ cells continued to increase in the RFA group. In contrast, in the surgery group, the number of CD4+ cells decreased in the first 7 postoperative days and then started to increase but did not reach the preoperative level. No significant difference was found at baseline, but after treatment, the number of CD4+ cells in the RFA group was higher than that in the surgery group (*p* < 0.01, Figure 1a). The number of CD8+ cells continued to decrease in the RFA group. In contrast, the number of CD8+ cells in the surgery group decreased in the first 7 postoperative days and then started to increase but did not reach the preoperative level. No significant difference was found at baseline, but the number of CD8+ cells in the RFA group was found to be higher on days 7 and 28 after treatment (Figure 1b). We calculated the CD4+/CD8+ ratio and found no difference at baseline, but we found that the ratio was higher in the RFA group than in the surgery group on days 7, 14, and 28 after treatment (Figure 1b). In the RFA group, the number of CD3+ cells was found to have kept on increasing on days 7 and 14 after treatment, whereas on day 28 after treatment, a slight reduction was found in the number of CD3+ cells, but it was still higher than the baseline value. In the surgery group, the number of CD3+ cells continued to decrease over the 28 postoperative days. No difference was found at baseline and on day 7 after treatment, but the number of CD3+ cells in the RFA group was higher than that in the surgery group on days 14 and 28 after treatment (*p* < 0.05, Figure 1d). The number of NK cells in both groups continued to increase, but the surgery group had a sharper curve than the RFA group (*p* < 0.01, Figure 1e)

### 3.3 Changes of immune cells in tumor tissues before and immediately after RFA

**Figure 2:**
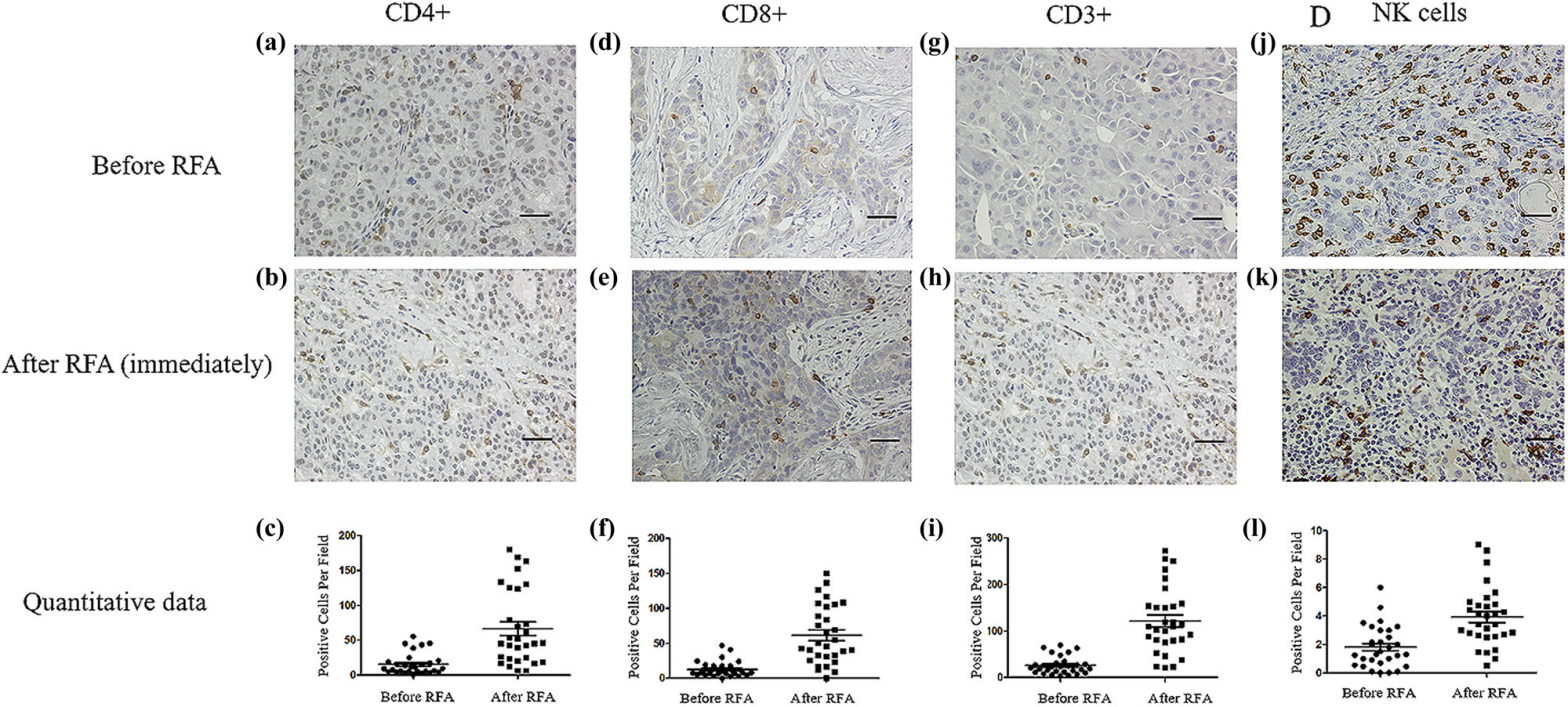
Immunohistochemical assay of immune cells in tumor tissues before and immediately after RFA. Before RFA: (a) CD4+ cells, (d) CD8+ cells, (g) CD3+ cells, and (j) NK cells. Immediately after RFA: (b) CD4+ cells, (e) CD8+ cells, (h) CD3+ cells, and (k) NK cells. Quantitative data: (c) CD4+ cells, (f) CD8+ cells, (i) CD3+ cells, and (l) NK cells. Data are presented as mean ± SEM. The brown cells are positive cells. Bar = 100 μm. RFA: radiofrequency ablation, CD: cluster of differentiation, NK: natural killer, SEM: standard error of the mean.

The result of the immunohistochemical assay of lymphocytes in the tumor tissues before and immediately after RFA is shown in Figure 2. We found that there were no morphological changes before and after treatment, but we found a change in the cellular density (Figure 2). There were statistically significant increments in the number of CD4+ cells (Figure 2c), CD8+ cells (Figure 2f), CD3+ cells (Figure 2i), and NK cells (Figure 2l) after RFA (*p* < 0.001).

## 4 Discussion

In this pilot study, we compared the changes of immune cells between the RFA and surgery groups at 7, 14, and 28 days after treatment. The changes of immune cells in tumor tissues immediately after RFA were also assessed. We found that RFA significantly increased circulating CD4+ cells, CD4+/CD8+, CD3+ cells, and NK cells compared to the preoperative levels, and cell levels were significantly higher in the RFA group than in the surgery group. Data of the immunohistochemical assay of tumor tissues showed a significant increase in the number of immune cells immediately after RFA. To the best of our knowledge, this is the first study to compare the number of circulating immune cells between patients who underwent RFA and patients who underwent conventional surgery and report changes of immune cells in tumor tissues after RFA. The results seem to imply that HCC patients treated with RFA might have less impact on the immunologic function in comparison with conventional surgery. These findings might be useful for the clinicians to make a decision on the selection of a better treatment for the HCC patients in terms of their actual conditions. We reckon that our findings will contribute to a better understanding of the immunological mechanisms associated with RFA.

The immune system is suppressed and immune function is impaired in patients with HCC, and this condition results in a state of tumor immune escape [18]. The immune system dysfunction in SHCC is based on cellular immunity. The changes of T cells subsets may be regarded as biomarkers of *in vivo* antitumor ability and immune system dysfunction [19]. It was reported that the characteristic changes of immune cells in SHCC include reduction in the number of CD4+ cells, reduction in the CD4+/CD8+ ratio, and increase in the number of CD8+ cells. Our data on PBMCs show that RFA significantly increases the number of circulating CD4+ cells (Figure 1a), and CD4+ levels in the RFA group were higher than those in the surgery group. Data on CD8+ cells show an opposite trend as RFA was found to significantly reduce the number of CD8+ cells (Figure 1b). It is noteworthy that, in the RFA group, the number of CD8+ cells maintained a downward trend over the observation period (28 days), but in the surgery group, the number of CD8+ cells decreased in the first 7 postoperative days and then began to increase thereafter. RFA significantly increased the CD4+/CD8+ ratio (Figure 1c). The upward trend of the CD4+/CD8+ ratio was maintained in the RFA group over 28 days, but in the surgery group, the ratio decreased in the first 7 days and then began to increase. During the onset of the tumor, both CD4+ and CD8+ cells are enriched at the tumor site. The roles of CD4+ cells are complicated and unclear. It has been documented that CD4+ cells are more plastic and play dual roles, namely antitumor or pro-tumor [20]. Yang et al. reported that the number of CD4+ cells significantly increased in the surrounding area of HCC tumor [21]. Fu et al. also found that the circulating CD4+ cells were significantly increased with the progression of HCC [22], which is in agreement with our data in both RFA and surgery groups. However, they also increased CD4+ cells that might be associated with high mortality and reduced the survival time in patients with HCC [22]. On the other hand, the roles of CD8+ cells seem to be simpler. It is known that CD8+ cells are the main drivers of the immune response in the tumor microenvironment [23]. It is a simple killer at the tumor site [20]. Abundant accumulation of CD4+ cells may result in a reduced infiltration of CD8+ cells in the HCC state [22]. Meanwhile, our results of immunohistochemistry at the tumor tissues found that both CD4+ and CD8+ cells significantly increased after RFA (Figure 2). Due to the complicated roles of CD4+ cells and interactions between CD4+ and CD8+ cells. We speculated that the numbers of circulating CD4+ and CD8+ cells are not only affected by the proliferation of these cells but also affected by the exhaustion of these cells. The reduction of circulating CD8+ cells might be a result of exhausted CD8+ cells, most of which are accumulated at the tumor site. The ratio of CD4+/CD8+ might be a better index to comprehensively understand the interaction between CD4+ and CD8+ cells, which plays a key role in the maintenance of the cellular immune balance. A previous study found that the circulating CD4+/CD8+ ratio is significantly lower in patients with HCC [24]. Despite our data indicated that the RFA group had a higher CD4+/CD8+ ratio; however, whether the higher CD4+/CD8+ ratio represents the better outcome requires further investigation. The changes of CD3+ cells showed completely opposite trends between the RFA and surgery groups (Figure 1d). The trend of NK cells was similar in both groups, but the NK cell level was higher in the surgery group than in the RFA group (Figure 1f). Since NK cells are associated with strong immune function, our data suggest that both RFA and surgery improve postoperative immune function in patients with SHCC. Our data on circulating immune cells show that RFA significantly improves immune function in patients with SHCC. To explain our findings, we propose the following underlying mechanisms: (1) immune inhibitors are released by tumor cells, which are inactivated by RFA, thereby reducing the level of immune inhibitors; (2) RFA may expose the surface of tumor cells or change the tumor antigens and subsequently activate cellular immune reaction against HCC; (3) RFA is reported to synthesize heat-shock proteins (HSPs) and particularly increase the release of HSP70. The release of HSPs may upregulate the expression of tumor-specific antigens, which contribute to immune system activation. Moreover, the discrepancy between the RFA and surgery groups indicates that these two treatment methods affect the immune system of patients differently. Hepatectomy is a relatively major surgery associated with high invasion and surgical injury. Many factors remarkably affect the immune system and may transiently suppress it. These factors include surgical processes, surgical injury, anesthesia, and postoperative response to surgical stress [25]. Our results show that, in the surgery group, the immune system was on a downward trend in the first 7 days after hepatectomy. Thereafter, the immune system began to recover, and clinicians should take note of these changes in the immune function.

There are several limitations to this study. First, we simply characterized the T cells as CD3+, CD4+, CD8+, and NK cells. However, T cells are classified into many subsets, which were characterized as “effector” T cells, “memory” T cells, and “regulatory” T cells. In addition, other immune cells apart from T and NK cells such as tumor-associated macrophages also critically influence the immune response in a tumor microenvironment. However, the present study did not investigate these functional subsets. Investigation of the subset should be included in our future investigations. Second, we did not conduct a long-term follow-up to observe the outcomes including the recurrence rates in each group, since it is well known that the recurrence rates (particularly local recurrences) are higher in the RFA group. Despite our data seem to indicate that the RFA group had a better immune function after treatment, the recurrence rate cannot be ignored during the selection of the therapies between RFA and surgery against HCC.

Our data on the immunohistochemical staining of tumor tissues show that the number of CD4+ cells, CD8+ cells, CD3+ cells, and NK cells increased immediately after RFA (Figure 2). The levels of CD4+ cells, CD3+ cells, and NK cells showed similar trends compared to those of PBMCs; however, the level of CD8+ cells showed a trend opposite to that of PBMCs. These data show that the changes of immune cells in tumor tissues are not completely consistent with those in the peripheral circulation. The pathological meaning of these changes requires further investigation.

## 5 Conclusion

In this study, we compared the changes of circulating immune cells between patients who underwent RFA and patients who underwent conventional hepatectomy. We also observed changes of immune cells in tumor tissues immediately after RFA. We found different trends in the RFA and surgery groups. In the RFA group, immune function after treatment kept on improving, whereas in the surgery group, postoperative immune function decreased over the first week and began to improve thereafter. Clinicians need to pay attention to these changes when they attempt to select a better treatment for patients with HCC. The data on tumor tissues were not completely consistent with that on PBMCs, and further investigation on the physiological meaning of these changes is required.
