# Erratum to “Changes of immune cells in patients with hepatocellular carcinoma treated by radiofrequency ablation and hepatectomy, a pilot study”

**DOI:** 10.1515/biol-2022-0007

**Published:** 2022-02-05

**Authors:** Zusheng Yu, Guowei Li, Hang Yu, Tetsuya Asakawa

**Affiliations:** Department of Hepatobiliary Surgery, The First People’s Hospital of Fuyang Hangzhou, Hangzhou, 311400, China; Department of Gastrointestinal Surgery, The First Affiliated Hospital, School of Medicine, Zhejiang University, Hangzhou, 310003, China; Department of Neurology, The Eighth Affiliated Hospital, Sun Yat-Sen University, Shennanzhong Road 3025, Shenzhen, Guangdong·Province, 518033, China; Department of Neurosurgery, Hamamatsu University School of Medicine, Handayama, Hamamatsu-city, Shizuoka, 4313192, Japan; Research Base of Traditional Chinese Medicine Syndrome, Fujian University of Traditional Chinese Medicine, Fuzhou 350122, China

In the published article Yu Z, Li G, Yu H, Asakawa T. Changes of immune cells in patients with hepatocellular carcinoma treated by radiofrequency ablation and hepatectomy, a pilot study. Open Life Sci. 2021;16(1):1002–9. 10.1515/biol-2021-0105, Figure 2h has been mistakenly inserted as a duplicate of Figure 2b. Upon realization, the authors requested an erratum to correct the mistake. The authors admit to the error and claim that this is an unintentional mistake that has nothing to do with academic misconduct and does not influence the conclusion of the publication. The raw data of the experiment have been shared with Journal Editors and are available from authors upon reasonable request.

**Figure 2:**
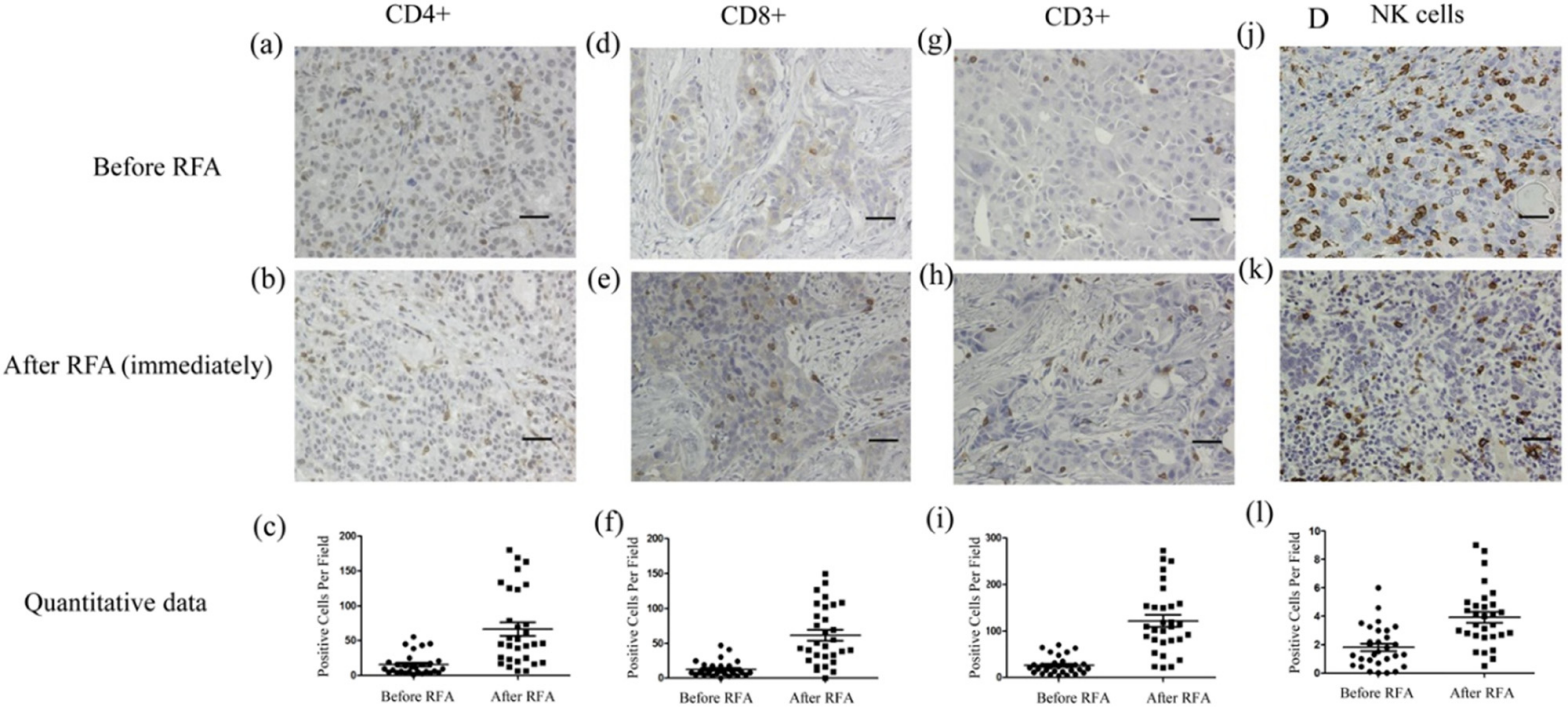
Immunohistochemical assay of immune cells in tumor tissues before and immediately after RFA. Before RFA: (a) CD4+ cells, (d) CD8+ cells, (g) CD3+ cells, and (j) NK cells. Immediately after RFA: (b) CD4+ cells, (e) CD8+ cells, (h) CD3+ cells, and (k) NK cells. Quantitative data: (c) CD4+ cells, (f) CD8+ cells, (i) CD3+ cells, and (l) NK cells. Data are presented as mean ± SEM. The brown cells are positive cells. Bar = 100 µm. RFA: radiofrequency ablation, CD: cluster of differentiation, NK: natural killer, SEM: standard error of the mean.

Figure 2 should be presented as follows:

The authors apologize to the Journal, readers, and their colleagues for the mistake and any inconvenience it caused.

